# Peer-Led Digital Health Lifestyle Intervention in a Low-Income Community at Risk for Cardiovascular Disease (MYCardio-PEER): Mixed Methods Development and Process Evaluation Study

**DOI:** 10.2196/77063

**Published:** 2025-11-12

**Authors:** Geok Pei Lim, Jamuna Rani Appalasamy, Badariah Ahmad, Kia Fatt Quek, Amutha Ramadas

**Affiliations:** 1 Jeffrey Cheah School of Medicine and Health Sciences Monash University Malaysia Subang Jaya Malaysia; 2 School of Pharmacy Monash University Malaysia Subang Jaya Malaysia

**Keywords:** digital health, peer support, lifestyle intervention, cardiovascular disease, low income

## Abstract

**Background:**

Low-income populations in Malaysia face significant barriers to cardiovascular disease (CVD) prevention, including limited access to health information, preventive services, and ongoing support for behavior change. Digital health technologies present scalable opportunities for promoting heart-healthy behaviors, especially when integrated with community-based peer support.

**Objective:**

This study aimed to describe the development and process evaluation of MYCardio-PEER (Peer-led digital health lifestyle intervention for CVD prevention in a low-income Malaysian community), designed to empower adults at risk for CVD through mobile technology and community-based peer engagement.

**Methods:**

MYCardio-PEER was developed using the Medical Research Council Framework for Developing and Evaluating Complex Interventions, which included evidence synthesis, theory application, intervention planning, and content design. A 2-round Delphi method was used to conduct content validation involving 10 experts and community panel members, assessing relevance, clarity, and cultural appropriateness. The final intervention was delivered over 8 weeks and included bite-sized educational videos and infographics, and peer-led interactive activities. A pre-post feasibility trial was conducted in Kulim, Kedah, with 30 adults identified as having moderate to high CVD risk. Outcome measures included anthropometric and clinical parameters, dietary intake, and physical activity. A process evaluation assessed participant adherence and program satisfaction, and correlations with health outcomes.

**Results:**

Participants (18/30, 60.0% male, mean age 58.2, SD 6.7 years) showed significant reductions in CVD risk score, systolic blood pressure, total cholesterol, and low-density lipoprotein cholesterol. Physical activity levels and selected dietary behaviors improved post intervention. Program adherence was high, with 82.4% of participants completing all peer-led sessions. Satisfaction with program content (85.7%) and peer leadership (96.0%) was strong. Participants described the content as accessible, engaging, and relevant to their daily lives.

**Conclusions:**

MYCardio-PEER was developed and content-validated as a peer-led digital health lifestyle intervention for a low-income community at risk of CVD. Process evaluation showed good feasibility and acceptability, with encouraging pre-post improvements in behaviors and selected health indicators. These findings suggest the potential of integrating peer support with culturally adapted digital content in community-based CVD prevention.

**Trial Registration:**

ClinicalTrials.gov NCT06408493; http://clinicaltrials.gov/study/NCT06408493

**International Registered Report Identifier (IRRID):**

RR2-10.1017/S1463423625000192

## Introduction

Cardiovascular disease (CVD) remains the leading global health concern as it contributes primarily to healthy life-years lost due to premature deaths and disability [1]. Similarly, in Malaysia, ischemic heart disease and stroke have dominated as the principal causes of death, recorded at 15.1% and 7.2%, respectively, of the total medically certified deaths in 2023 [2]. Concomitantly, the National Health and Morbidity Survey 2023 revealed that nearly 2.3 million adults in Malaysia live with a cluster of 3 noncommunicable diseases, including diabetes, hypertension, high cholesterol, and obesity, which are major risk factors for CVD [3]. Per the World Health Organization’s report that 75% of CVD deaths occur in low- and middle-income countries [4], the prevalence of CVD and its associated risk factors also disproportionately affected the low-income populations in Malaysia [5,6].

Previous studies have shown that low-income groups are exposed to a higher risk of CVD-related morbidities due to their increased likelihood of engaging in unhealthy behaviors, limited social protection, and lack of access to preventive health care [7]. In Malaysia, the “low-income” population is commonly defined as the bottom 40% (B40) of households, based on national income distribution. In Kedah, a semi-urban state, this includes households earning less than RM 3710 (US $878.31) per month or those receiving government aid under B40-targeted programs [8]. Besides systemic socioeconomic disadvantages, the modifiable behavioral risk factors had been identified as unhealthy dietary patterns, including low intake of fruits and vegetables, high intake of ultra-processed foods and calorie-dense local foods [9], physical inactivity [10], and being a smoker [11]. While mass screening and health initiatives targeting low-income communities such as PeKa B40 have been promoted by the Malaysian government [12], there remains a lack of culturally specified lifestyle interventions to sustain daily health behaviors for managing and preventing CVD risk factors among this vulnerable population.

In attempts to reduce the CVD burden, various lifestyle interventions for primary prevention have been conducted worldwide. More recently, peer-led interventions have emerged as a promising approach to tackling CVD risk, particularly in resource-limited settings [13]. Peer support is pivotal in fostering and sustaining healthy behaviors and managing chronic diseases by providing a sense of community, ongoing emotional support, and motivation to overcome daily challenges [14]. Notably, group-oriented and peer-based approaches were found to enhance enjoyment and engagement in healthy lifestyles among individuals with lower socioeconomic backgrounds [15]. These interventions can be particularly impactful in low-income communities, leveraging local networks to promote trust and engagement, thus leading to healthier lifestyles and better cardiovascular outcomes. However, their application in Malaysia still needs to be improved, with gaps in research exploring culturally tailored peer-support strategies to mitigate CVD risk factors [16].

With the widespread use of smartphones and internet access at an unprecedented rate, 98.4% of the Malaysian adult population as of 2023, peer-led interventions for CVD prevention can effectively integrate digital health approaches to enhance their impact [17,18]. Digital connectivity has opened a broader reach, supplementing traditional in-person interventions and the convenience and flexibility that can motivate individuals to engage better with and sustain healthy lifestyle programs [19,20]. In our study setting, although participants were classified as low-income (B40), most had completed secondary education and owned internet-connected devices. However, variation in digital confidence and health literacy remains a challenge. Delivering videos and infographics via mobile messaging platforms offers an accessible and engaging method for health education, educating individuals about modifiable risk factors for CVD and providing actionable tips on lifestyle modifications [21]. The combination of peer support and digital technologies reinforces information in a relatable and easily digestible format, making complex concepts simpler to understand and remember, and leverages the influence of peer networks to encourage the adoption and maintenance of heart-healthy behaviors.

A scoping review of existing interventions in Malaysia accentuates the limited focus on combining peer-led strategies with digital health tools for the primary prevention of CVD in marginalized populations [22]. There is an urgent need to develop preventive strategies addressing health inequality and optimizing resource allocation to reduce the disproportionate burden of CVD in Malaysia’s low-income communities. Hence, this study aimed to describe the development and process evaluation of the MYCardio-PEER program, a peer-led digital health lifestyle intervention for primary prevention of CVD in a low-income community. The process evaluation focused on program delivery, participant adherence, satisfaction, and pre-post changes in selected health indicators among intervention participants. The comparative effectiveness of the intervention using a quasi-experimental design with a control group is reported in a separate publication [23].

## Methods

### Study Design and Setting

This study is part of the main quasi-experimental study with an overall study duration of 20 weeks, including participant recruitment, baseline assessment, the 8-week postintervention assessment, and 20-week post-follow-up assessment [23]. This paper focuses specifically on the development and feasibility assessment of the 8-week peer-led intervention component. The study adopted a pre-post design without a control group, suitable for evaluating the feasibility, acceptability, and short-term effects of the intervention. The intervention was implemented in Lunas, Kulim, and Kedah, in collaboration with the Kamakshi Welfare and Social Association, a grassroots community-based organization.

### Intervention Development

**Figure 1:**
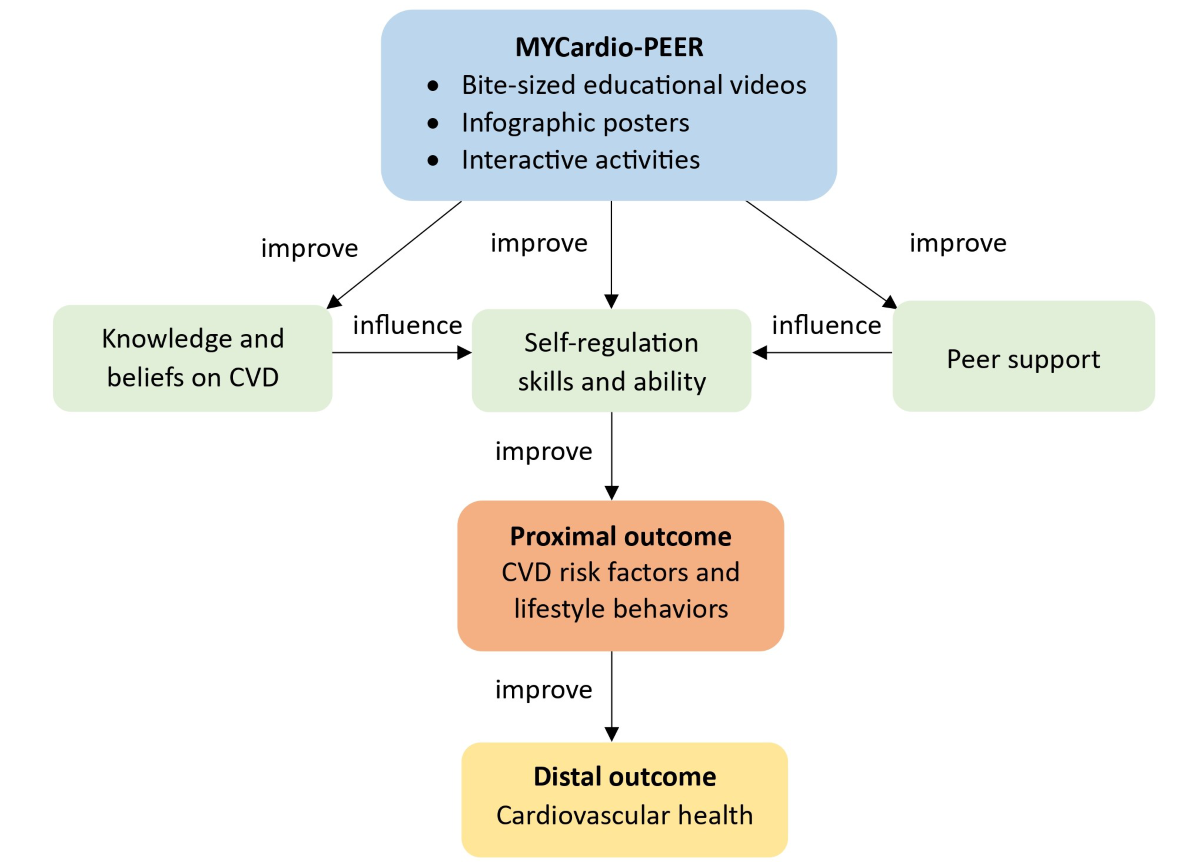
Theoretical framework of MYCardio-PEER. CVD: cardiovascular disease.

The MYCardio-PEER was developed following the Medical Research Council (MRC) Framework for Developing and Evaluating Complex Interventions (MRC framework) [24,25]. This study focused on the first phase of the updated MRC framework, which involved developing a complex intervention. Four key dynamic actions of intervention development were involved: reviewing published research evidence, drawing on existing theories, planning the development process, and designing and refining the intervention [25]. The theoretical framework of MYCardio-PEER is depicted in Figure 1. The intervention aimed to promote heart-healthy behaviors through peer support, digital content delivery, and culturally appropriate strategies. Full details of the intervention development process are presented in Multimedia Appendix 1 [13,17,21,26-32].

### Intervention Content Design and Validation

The content for the 8-week MYCardio-PEER program was outlined based on the evidence gathered, behavioral change matrix, and program development guide. The delivery formats were decided based on the information’s complexity, handiness, and experiential nature [33,34]. Delivery strategies include bite-sized videos, infographic posters, and peer-led activities.

The infographic posters and motion videos were created using Canva (Canva Pty Ltd, Sydney, Australia). The expert interview videos and specific footage were captured using an iPhone XR (Apple Inc, 2018). These videos were then processed and edited using the CapCut mobile app (Bytedance Ltd). Peer-led activities were planned and compiled as a health progress booklet. All the content was created in English and Malay languages.

### Content Validation Process

The content validation of the MYCardio-PEER program was conducted using the Educational Content Validation Instrument in Health [35] among 10 expert panels. The expert panel consisted of 5 health care professionals, including a medical doctor, 2 pharmacists, a nutritionist, a dietitian, and 5 local community members who were selected as peer leaders for the program. Involving peer leaders in the validation process ensured that delivery strategies and content were shaped by individuals who shared lived experiences with the target population. The validation instrument included 18 items divided into 3 key domains: objectives, structure/presentation, and relevance. The items were rated on a scale where 0 indicated disagreement, 1 indicated partial agreement, and 2 indicated strong agreement. A comment section was provided for each item, allowing panels to justify their ratings and suggest improvements. All feedback was carefully reviewed and incorporated into revisions of the content. A 2-round Delphi method was used to refine the content until consensus was reached [36]. Community feedback included suggestions to simplify technical terms, improve visual design, and enhance cultural relevance. Professionals focused on clarity, accuracy, and alignment with guidelines. Revisions were made between rounds, including language simplification and graphic adjustments. The final content was developed in both English and Malay to ensure accessibility.

### Content Validation Analysis

The results were analyzed using the Content Validity Index, which quantifies the degree of agreement among experts on the relevance of each item. First, the relevance ratings by 10 panels were entered in Microsoft Excel. Items ranked “disagree” were designated “0,” while “partially agree” and “strongly agree” were designated “1.” Then, the item-level Content Validity Index and scale-level Content Validity Index were computed based on the calculation methods provided by Yusoff [37]. For the content to achieve a satisfactory validity rating, it must reach a minimum agreement level of 80%.

### Participants and Recruitment

The developed and validated content of MYCardio-PEER was tested for its feasibility via a pre-post trial design. Local community members were recruited if they were confirmed to have a moderate to high risk for CVD based on the Framingham General CVD Risk Score (FRS) [26]; had a mobile device connected to the internet; were able to understand the Malay language; and were willing to commit to 8 weeks of intervention. Individuals with pre-existing severe medical conditions (eg, uncontrolled diabetes and recent cardiac events) were excluded to minimize health risks and reduce variability in outcome measures unrelated to the intervention. Participants’ informed consent was obtained before the study commencement. The intervention participants were divided into 5 groups, each consisting of 6 members, based on their peer connectivity.

A total of 5 peer leaders were recruited through personal interviews and selected based on leadership qualities, communication skills, and commitment to the program’s objectives. They were screened for moderate to high CVD risk and chosen from the same community to ensure shared experiences with participants. Kamakshi Welfare and Social Association provided recommendations, and the final selection involved discussions and interviews. The leaders then completed a 2-day training to develop facilitation skills and support behavior change.

Following recruitment and peer leader training, participants completed baseline assessments and began the 8-week intervention. Digital materials were delivered via the WhatsApp group chat and complemented with both digital and in-person peer-led interactive activities.

### Data Collection and Outcome Measures

Changes in anthropometric measures, FRS, CVD-related biomarkers, physical activity levels, and dietary intakes were recorded at baseline and 8 weeks postintervention using a structured questionnaire (Multimedia Appendix 2). Body height and weight were measured using the SECA 777 Column Scale. BMI was calculated as weight in kilograms divided by the square of height in meters. Waist circumference was measured using the SECA 201 Ergonomic Circumference Measuring Tape. Blood pressure was assessed using an Omron Automatic Blood Pressure Monitor HEM-7120. The finger-prick method was used to measure fasting blood glucose using the Autocode Glucometer and total cholesterol (TC), high-density lipoprotein cholesterol, low-density lipoprotein cholesterol (LDL-C), and triglyceride using the CardioCheck PA Analyzer System. FRS was calculated based on age, sex, levels of high-density lipoprotein cholesterol and TC, systolic blood pressure (SBP), smoking habits, and diabetes.

Physical activity levels were assessed using the International Physical Activity Questionnaire [38]. According to the International Physical Activity Questionnaire scoring protocol, responses were converted to metabolic equivalent task minutes per week. Dietary intakes were assessed using the short food frequency questionnaire and a 2-day 24-hour dietary recall [39].

### Process Evaluation

The process evaluation of MYCardio-PEER was conducted at the end of the 8-week program to assess the participants’ adherence, satisfaction, and perceived acceptability, accessibility, and engagement with the intervention. Peer attendance in completing the peer-led activities was used to determine program adherence.

A structured, self-administered questionnaire was used to evaluate participants’ satisfaction with the program content and delivery and peer leadership. The instrument was adapted from a previously validated tool developed by Mahadzir et al [40]. Satisfaction with program content was assessed across 5 items: structure, content relevance, delivery style, and engagement strategies of the program. Peer leadership was assessed based on the peer leader’s knowledge, communication, encouragement, and overall supportiveness. The responses were recorded using a Likert scale with scores ranging from 1 (strongly disagree) to 5 (strongly agree). The sum of the responses for each domain totaled 100%, with higher scores indicating higher satisfaction. The questionnaire on program satisfaction is presented in Multimedia Appendix 3.

In addition, participants were invited to provide open-ended feedback on their experiences, including what they found helpful or challenging, their comfort using mobile-based content, and their views on peer interactions. These comments were reviewed thematically to identify common perceptions related to program accessibility, acceptability, and engagement.

### Data Analysis

Descriptive statistics were used to describe the participants’ demographic characteristics. The normality of all continuous variables was determined using the Shapiro-Wilk test. A paired sample *t* test and Wilcoxon signed-rank test were performed to compare the changes in anthropometric measures, clinical measures, physical activity levels, and dietary intakes between baseline and postintervention. Spearman rho (ρ) was used to assess the correlation between satisfaction towards content and peer leadership with changes in the outcome parameters. Statistical analysis was performed with SPSS Statistics (version 27.0; IBM Corp) with statistical significance set at *P*<.05 (2-tailed).

### Ethical Considerations

Ethical approval for this study was obtained from the Monash University Human Research Ethics Committee (MUHREC ID: 36440). All participants provided written informed consent prior to enrollment in the study. The consent form included information regarding the study purpose, procedures, potential risks and benefits, confidentiality protections, and the voluntary nature of participation. Participants were informed that they could withdraw from the study at any time without penalty or loss of benefits.

Data were collected and stored in a secure database, and all records were anonymized using participant codes to ensure privacy and confidentiality. Only deidentified data were used in the analyses. All participants received a small honorarium of RM 150 (US $35.51) as appreciation for their time and commitment to the study. This compensation was not intended to influence participation and was approved by the ethics review board. No identifiable images of participants are included in this manuscript or supplementary materials.

## Results

### Intervention Content Design and Validation

The MYCardio-PEER program consisted of 8-weekly modules that consisted of bite-sized videos, infographic posters, and a health progress booklet for peer-led activities. Each week focused on a specific heart-health topic, including physical activity, blood pressure control, healthy eating, and stress management. Table 1 outlines the weekly intervention topics and materials, along with the corresponding content validity ratings assessed by expert panels. The scale-level Content Validity Index, using both the average and universal agreement methods, exceeded 0.80, indicating satisfactory agreement on content relevance and clarity. Items that did not initially reach a Content Validity Index of 1.00 were revised and re-evaluated in the second round.

Qualitative feedback was instrumental in improving the intervention materials. For example, complex terminology such as “trans-fat” was replaced with simplified explanations, and infographic layouts were adjusted to improve readability by increasing font size and visual contrast. These refinements were made to enhance the cultural appropriateness, visual clarity, and overall usability of the materials for the target population.

**Table 1:** Content validation ratings and weekly topic outline of MYCardio-PEER materials by 10 expert panel members.

Week, topic, and content			S-CVI/Ave^a^	S-CVI/UA^b^
**Week 1: Are you at risk of heart disease?**				
	1.1 Bite-sized video to explain the structure of the heart, its functions, and signs of heart attack.	0.99		0.89
	1.2 Bite-sized video on CVD^c^ risk factors.	0.98		0.83
	1.3 Infographic poster on biomarkers reading.	0.99		0.94
**Week 2: Say YES to physical activity**				
	2.1 Bite-sized video to explain the benefits and recommendations of physical activity.	1.00		1.00
	2.2 Bite-sized video to describe types of physical activity and tips.	1.00		1.00
	2.3 Infographic poster on the physical activity pyramid.	0.99		0.89
**Week 3: How to maintain a healthy weight?**				
	3.1 Bite-sized video to explain calories, serving size, food pyramid and healthy plate.	1.00		1.00
	3.2 Bite-sized video on weight control tips.	1.00		1.00
	3.3 Infographic poster on lower calorie food swap.	1.00		1.00
**Week 4: Control your blood pressure**				
	4.1 Bite-sized video to explain hypertension and its complications.	0.99		0.94
	4.2 Bite-sized video on healthy diet and lifestyle habits for maintaining healthy blood pressure.	0.99		0.94
	4.3 Infographic poster on lower sodium food swap and reading food labels for sodium.	0.99		0.94
**Week 5: Keep your cholesterol in check**				
	5.1 Bite-sized video on cholesterol and how it affects the body.	0.99		0.89
	5.2 Bite-sized video on a heart-healthy diet.	0.99		0.94
	5.3 Infographic poster on reducing fat intake.	0.99		0.94
**Week 6: The not-so-sweet truths**				
	6.1 Bite-sized video on diabetes, symptoms, and complications.	0.99		0.94
	6.2 Bite-sized video on choosing carbohydrates foods.	1.00		1.00
	6.3 Infographic posters on lower sugar food swap and reading food labels for sugar.	1.00		1.00
**Week 7: Enjoy living smoke-and-stress-free**				
	7.1 Bite-sized video on harmful effects of smoking and tips to quit smoking.	0.99		0.94
	7.2 Infographic poster on overcoming nicotine withdrawal symptoms.	0.98		0.83
	7.3 Bite-sized video on stress and its management.	0.99		0.89
**Week 8: Practical tips for heart-healthy meals**				
	8.1 Bite-sized video on time-saving and healthy meal preparation.	1.00		1.00
	8.2 Bite-sized video on healthier options when eating out.	0.99		0.89
	8.3 Infographic poster on choosing packaged foods.	0.99		0.94
**Weeks 1-8: Health progress booklet**				
	Peer-led activities.	0.99		0.94

^a^S-CVI/Ave: scale-level Content Validity Index based on the average method.

^b^S-CVI/UA: scale-level Content Validity Index based on the universal agreement method.

^c^CVD: cardiovascular disease.

### Study Participants

Thirty participants with moderate-to-high risk of CVD were enrolled in the feasibility trial, with a mean age of 58.2 (SD 6.7), of which 60.0% (n=18) participants were males. Around half of the participants (17/30, 56.7%) worked with a monthly household income of RM 1800.31 (SD 868.75), approximately US $400.41 (SD 193.22). Most participants were married (26/30, 86.7%) and attained secondary education (25/30, 83.3%).

### Changes in Outcome Measures

While no significant changes were observed for anthropometric measures, significant changes were found in clinical measures. Despite the median FRS remained unchanged at 17.2, there was a significant difference in the distribution of scores between time points (IQR –1.3 to 0; z=2.090; *P*=.04). Significant reductions in SBP (median difference –2.8, IQR –9.8 to 0; z=–3.876; *P*<.001), TC (median difference –0.16, IQR –0.74 to 0; z=–2.813; *P*=.005), and LDL-C (median difference –0.14, IQR –0.71 to 0.03; z=–2.362; *P*=.02) were observed.

The total physical activity levels were significantly increased postintervention (mean difference 217.0, SD 489.9; t=2.426; *P*=.02). The intervention participants increased their fruits intake from 0.7 (IQR 0.3 to 2.2) to 1.1 (IQR 0.8 to 1.5) servings per day *(z*=3.305; *P*<.001), while the daily intakes of fish and seafood increased from 0.4 (IQR 0.3 to 1.1) to 0.7 (IQR 0.5 to 1.1; *z*=2.237; *P*=.03). As for nutrient intakes, there were significant reductions in energy (median difference –231.64, IQR –723.54 to –15.42; z=–4.535; *P*<.001) and sodium intakes (median difference –146.79, IQR –451.79 to 50.02; z=–2.149; *P*=.03). Notably, potassium (median difference 95.99, IQR 6.54 to 283.14; z=2.808; *P*=.005), fiber (median difference 1.24, IQR 0.20 to 3.85; z=4.103; *P*<.001), and vitamin C (median difference 3.59, IQR 0.79 to 34.94; z=3.527; *P*<.001) intakes were increased postintervention. These dietary and nutrient outcomes reflect specific behavioral targets of the MYCardio-PEER intervention, which aimed to promote healthier food choices and support practical changes in daily eating habits through peer-led activities and digital content. Table 2 shows the changes in the participants’ outcome measures.

**Table 2:** Changes in outcome measures before and after the MYCardio-PEER intervention (n=30). Wilcoxon signed-rank test was used for comparing medians, and the paired samples t test was used for comparing means.

		Baseline		Postintervention		Change		Test statistics		*P* value
**Anthropometric measures, median (IQR)**										
	Weight (kg)	67.50 (62.60 to 75.01)	67.75 (60.93 to 75.01)		0 (–1.19 to 0.03)		–0.900		.37	
	BMI (kg/m^2^)	26.61 (23.91 to 30.92)	25.86 (23.99 to 30.18)		0 (–0.47 to 0.01)		–0.805		.42	
	Waist circumference (cm)	95.55 (89.19 to 102.36)	95.55 (89.55 to 103.05)		0 (–1.15 to 0)		–1.250		.21	
**Clinical measures, median (IQR)**										
	FRS^a^	17.2 (13.2 to 25.3)	17.2 (13.2 to 21.6)		0 (–1.3 to 0)		2.090		.04	
	SBP^b^ (mmHg)	135.3 (132.1 to 141.1)	134.0 (125.0 to 139.5)		–2.8 (–9.8 to 0)		–3.876		<.001	
	DBP^c^ (mmHg)	84.5 (76.5 to 90.4)	82.8 (74.8 to 89.0)		–0.5 (–4.0 to 1.1)		−1.500		.13	
	FBG^d^ (mmol/L)	5.9 (5.5 to 7.4)	6.2 (5.5 to 7.4)		–0.2 (–0.7 to 0.4)		–0.943		.35	
	TC^e^ (mmol/L)	3.87 (3.28 to 4.91)	3.62 (3.08 to 3.96)		–0.16 (–0.74 to 0)		–2.813		.005	
	HDL-C^f^ (mmol/L)	1.02 (0.91 to 1.18)	1.10 (0.98 to 1.18)		0.01 (–0.03 to 0.10)		1.423		.16	
	LDL-C^g^ (mmol/L)	2.16 (1.66 to 2.87)	2.00 (1.43 to 2.23)		–0.14 (–0.71 to 0.03)		–2.362		.02	
	TG^h^ (mmol/L)	1.25 (0.82 to 1.54)	1.14 (0.95 to 1.39)		0 (–0.30 to 0.06)		–1.080		.28	
**Physical activity level, mean (SD)**										
	METs^i^-minutes/week	1021.1 (862.6)	1238.1 (831.5)		217.0 (489.9)		2.426		.02	
**Dietary intakes, median (IQR)**										
	Grains (servings/day)	3.7 (2.7 to 4.6)	3.8 (2.8 to 4.4)		–0.1 (–0.6 to 0.2)		–0.876		.38	
	Fruits (servings/day)	0.7 (0.3 to 1.2)	1.1 (0.8 to 1.5)		0.1 (0 to 0.5)		3.305		<.001	
	Vegetables (servings/day)	1.1 (0.7 to 2.2)	1.6 (1.0 to 2.6)		0 (–0.1 to 0.8)		1.503		.13	
	Fish and seafood (servings/day)	0.4 (0.3 to 1.1)	0.7 (0.5 to 1.1)		0 (0 to 0.4)		2.237		.03	
	Processed foods (servings/day)	1.6 (0.5 to 2.3)	1.4 (0.7 to 2.0)		0 (–0.2 to 1.2)		–0.942		.35	
**Nutrient intakes, median (IQR)**										
	Energy (kcal)	1867.00 (1621.62 to 2176.87)	1444.32 (1210.87 to 1692.68)		–231.64 (–723.54 to –15.42)		–4.535		<.001	
	Protein (g/1000 kcal)	41.19 (37.68 to 46.81)	43.47 (36.48 to 49.53)		–0.40 (–3.56 to 5.15)		–0.072		.94	
	Carbohydrate (g/1000 kcal)	120.00 (113.09 to 134.01)	133.17 (117.74 to 146.78)		1.37 (–1.10 to 23.86)		1.656		.10	
	Fat (g/1000 kcal)	38.40 (31.75 to 43.09)	31.53 (28.37 to 39.06)		–0.62 (–8.42 to 1.25)		–1.635		.10	
	Saturated fat (g/1000 kcal)	5.17 (3.58 to 8.84)	4.61 (2.61 to 5.87)		–0.33 (–2.13 to 0.34)		–1.944		.05	
	Monounsaturated fat (g/1000 kcal)	5.84 (3.26 to 8.04)	4.79 (3.17 to 6.91)		–0.26 (–1.61 to 0.29)		–1.512		.13	
	Polyunsaturated fat (g/1000 kcal)	5.83 (3.23 to 7.25)	6.04 (4.20 to 8.06)		0.16 (–0.88 to 1.40)		0.812		.42	
	Sodium (mg/1000 kcal)	1428.73 (937.99 to 1593.88)	1018.88 (780.30 to 1406.87)		–146.79 (–451.79 to 50.02)		–2.149		.03	
	Potassium (mg/1000 kcal)	765.54 (578.13 to 935.42)	917.22 (706.48 to 1153.76)		95.99 (6.54 to 283.14)		2.808		.005	
	Dietary fiber (g/1000 kcal)	1.74 (1.11 to 3.98)	3.85 (2.38 to 6.91)		1.24 (0.20 to 3.85)		4.103		<.001	
	Sugar (g/1000 kcal)	13.23 (7.84 to 23.74)	12.73 (5.86 to 21.10)		–1.01 (–3.45 to 2.17)		–1.203		.23	
	Vitamin C (mg/1000 kcal)	27.68 (10.95 to 45.26)	44.34 (12.05 to 85.89)		3.59 (0.79 to 34.94)		3.527		<.001	

^a^FRS: Framingham General CVD Risk Score.

^b^SBP: systolic blood pressure.

^c^DBP: diastolic blood pressure.

^d^FBG: fasting blood glucose.

^e^TC: total cholesterol.

^f^HDL-C: high-density lipoprotein cholesterol.

^g^LDL-C: low-density lipoprotein cholesterol.

^h^TG: triglyceride.

^i^MET: metabolic equivalents.

### Process Evaluation

Program adherence among the 30 participants was high, with an average peer attendance of 82.4% for completing the peer-led activities. Trends in weekly peer session attendance during the MYCardio-PEER intervention are summarized in Table 3. Program adherence each week was at least 80.0%. Peer-led activities in week 8 were the most popular, achieving the highest attendance rate of 93.8%, while activities in week 6 were the least popular, with an attendance rate of 80.0%. Overall, participants’ satisfaction with the program was high, with a median content satisfaction score of 85.7% (IQR 80.0 to 97.1). Satisfaction with peer leadership was even higher, with a median score of 96.0% (IQR 85.0 to 100.0).

**Table 3:** Trends in weekly peer session attendance during the MYCardio-PEER intervention.

Week	Values, n (%)
1	27 (90.0)
2	26 (86.7)
3	25 (83.3)
4	27 (90.0)
5	25 (83.3)
6	24 (80.0)
7	27 (90.0)
8	30 (100.0)

In addition to quantitative ratings, qualitative feedback from participants provided insights into the program’s acceptability and accessibility. Most participants reported that the content was easy to understand and culturally relevant. They appreciated the flexibility of accessing videos via mobile phones, especially during their own time. Several participants highlighted that they felt more comfortable learning from peers who spoke their language and shared similar health concerns. Engagement was further enhanced by the interactive nature of the peer-led sessions, which participants described as encouraging and motivating. A few participants noted initial difficulties with navigating digital content, which were overcome with peer assistance and simple design features. These findings support the high levels of acceptability, accessibility, and engagement observed across the program.

### Correlation Between Process Evaluation and Study Outcomes

There was a moderate, statistically significant positive correlation between program adherence and the increase in carbohydrate (ρ=0.410; *P*=.02) and vitamin C (ρ=0.460; *P*=.01) intake, as well as reductions in fat (ρ=–0.388; *P*=.03) and sugar intake (ρ=0.505; *P*=.004). Content satisfaction was correlated with reductions in TC (ρ=–0.375; *P*=.04) and LDL-C (ρ=–0.379; *P*=.04), as well as with increases in fruit consumption (ρ=0.422; *P*=.02) and potassium intake (ρ=0.383; *P*=.04). Significant correlations were also found between satisfaction with peer leadership and reductions in energy consumption (ρ=0.447; *P*=.01) and the increase in vitamin C intake (ρ=–0.396, *P*=.03). The correlation between program adherence, content satisfaction, and satisfaction with peer leadership, and changes in outcome measures are presented in Table 4.

**Table 4:** Correlation between program adherence, content satisfaction, and satisfaction with peer leadership, and changes in outcome measures.

Changes in measures		Program adherence			Content satisfaction		Satisfaction with peer leadership	
		ρ^a^	*P* value	ρ^a^		*P* value	ρ^a^	*P* value
**Anthropometric measures**								
	Weight (kg)	–0.207	.21	0.061		.75	0.045	.82
	BMI (mg/m^2^)	–0.197	.30	0.037		.85	0.114	.55
	Waist circumference (cm)	–0.115	.55	–0.088		.64	–0.014	.94
**Clinical measures**								
	FRS^a^	–0.188	.32	–0.399		.03	0.088	.64
	SBP^b^ (mmHg)	0.072	.71	–0.190		.32	0.030	.88
	DBP^c^ (mmHg)	0.323	.08	–0.039		.84	0.206	.28
	FBG^d^ (mmol/L)	0.411	.02	–0.320		.09	–0.095	.62
	TC^e^ (mmol/L)	0.143	.45	–0.375		.04	0.213	.26
	HDL-C^f^ (mmol/L)	–0.133	.49	–0.179		.34	–0.020	.92
	LDL-C^g^ (mmol/L)	–0.024	.90	–0.379		.04	0.154	.42
	TG^h^ (mmol/L)	–0.008	.97	–0.038		.84	0.285	.13
**Physical activity level**								
	METs^i^-minutes/week	0.153	.42	–0.169		.37	–0.079	.68
**Dietary intakes**								
	Grains (servings/day)	–0.032	.87	0.097		.61	0.052	.79
	Fruits (servings/day)	0.058	.76	0.422		.02	–0.291	.12
	Vegetables (servings/day)	0.326	.08	–0.062		.74	–0.192	.31
	Fish and seafood (servings/day)	–0.066	.73	–0.102		.59	–0.099	.60
	Processed foods (servings/day)	–0.128	.50	–0.052		.79	–0.102	.59
**Nutrient intakes**								
	Energy (kcal)	–0.327	.08	–0.280		.13	0.447	.01
	Protein (g/1000 kcal)	–0.006	.97	–0.119		.53	0.016	.93
	Carbohydrate (g/1000 kcal)	0.410	.02	0.142		.46	0	>.99
	Fat (g/1000 kcal)	–0.388	.03	0.012		.95	–0.128	.50
	Saturated fat (g/1000 kcal)	–0.015	.94	0.063		.74	0.289	.12
	Monounsaturated fat (g/1000 kcal)	–0.208	.27	–0.153		.42	–0.059	.76
	Polyunsaturated fat (g/1000 kcal)	–0.173	.36	0.125		.51	–0.102	.59
	Sodium (mg/1000 kcal)	0.009	.96	–0.132		.49	0.111	.56
	Potassium (mg/1000 kcal)	0.281	.13	0.383		.04	–0.168	.37
	Dietary fiber (g/1000 kcal)	0.190	.32	0.191		.31	–0.350	.06
	Sugar (g/1000 kcal)	0.505	.004	0.074		.70	–0.006	.98
	Vitamin C (mg/1000 kcal)	0.460	.01	0.229		.22	–0.396	.03

^a^FRS: Framingham General CVD Risk Score.

^b^SBP: systolic blood pressure.

^c^DBP: diastolic blood pressure.

^d^FBG: fasting blood glucose.

^e^TC: total cholesterol.

^f^HDL-C: high-density lipoprotein cholesterol.

^g^LDL-C: low-density lipoprotein cholesterol.

^h^TG: triglyceride.

^i^MET: metabolic equivalents.

## Discussion

### Principal Findings

This study outlined a systematic and structured process of the development, content validation, and process evaluation of a peer-led digital health lifestyle intervention to prevent CVD in a low-income community (MYCardio-PEER). Guided by the MRC framework, the development used an evidence-based approach incorporating current research evidence and sound theoretical foundations. The local cultural context was taken into consideration through intervention mapping and content validation at the community level. These elements informed the design of a digitally-enhanced, person-centered lifestyle intervention tailored to the low-income setting, ensuring that the intervention addressed the unique challenges faced by this community, such as limited access to preventive health care resources.

The intervention included bite-sized educational videos, infographic posters, and interactive activities targeting knowledge, nutrition, and lifestyle behaviors for CVD prevention. Digital video is a highly effective tool for simplifying complex topics with captivating visuals, enabling learners to progress at their own pace, and often providing an efficient and scalable way to increase health awareness [41]. Meanwhile, infographic posters that exhibit actionable tips could better educate and engage the audience [21]. Besides improving knowledge and promoting health behaviors via visual media, the maintenance of healthy behaviors requires peer support for ongoing emotional support and motivation, especially among individuals with lower socioeconomic backgrounds [14,15].

For newly developed content, the validation process ensures that it aligns with evidence-based facts, is easily understood, and is relevant to low-income individuals at risk of CVD [42]. To achieve this, MYCardio-PEER adopted a co-design approach that actively involved both health care professionals and local community members in reviewing every video, infographic poster, and interactive activity. Engaging experts ensures scientific accuracy, while input from individuals with lived experiences could enhance cultural and contextual relevance, making the content more engaging and actionable [43]. With meticulous preparation before content creation, MYCardio-PEER achieved a satisfactory level in the first round of the Delphi assessment. Nonetheless, qualitative feedback was incorporated to refine the layout and enhance the comprehensiveness of the content, reflecting the iterative nature of co-design in digital health interventions.

MYCardio-PEER significantly reduced SBP and TC, resulting in improved FRS. These outcomes are attributable to increased physical activity levels facilitated by peer coaching and social connection [44]. Additionally, mindful and healthful dietary choices may provide nutrients with cardioprotective effects [45]. Our findings showed that participants significantly increased their intake of fruits and vegetables, thereby boosting fiber and vitamin C consumption while reducing total energy and sodium intake. The reduction in energy intake may reflect improved portion control or reduced consumption of calorie-dense foods encouraged by the intervention. However, the postintervention mean intake of approximately 1444 kcal/day is lower than national recommendations for adults and may indicate potential underreporting, a common limitation of self-reported dietary assessments. Nevertheless, the inclusion of these detailed dietary and nutrient outcomes provides insight into the behavioral pathways through which cardiovascular risk may be reduced.

These positive changes align with the Integrated Theory of Behavior Change, which emphasizes the role of knowledge, self-regulation, and social support in sustaining behavior modifications. The peer-led structure of MYCardio-PEER enhanced self-efficacy and motivation, enabling participants to adopt and maintain healthier lifestyle habits. By integrating behavioral strategies such as goal setting, self-monitoring, and peer reinforcement, the intervention effectively supported sustainable improvements in physical activity and dietary intake. The peer-led format may have been particularly effective in this setting, where shared lived experiences and cultural familiarity helped foster trust and accountability. Taken together, these findings support MYCardio-PEER as a feasible and acceptable initiative to mitigate CVD risk through improved dietary and lifestyle behaviors.

Furthermore, the high program adherence observed highlights the effectiveness of the MYCardio-PEER intervention in engaging participants and sustaining their involvement. This is an encouraging indicator of the program’s feasibility and acceptance among the target group. The variability in attendance rates, with week 8 achieving the highest participation and week 6 the lowest, could indicate that participants appreciated the practical tips provided for preparing healthy meals and creating meal plans. In contrast, the lower attendance might have been due to the more challenging task of reading food labels and recording their reduced sugar actions. Identifying and replicating the aspects of week 8 that resonated with participants could enhance attendance and engagement in future implementations. Meanwhile, the high satisfaction scores reflect the program’s success in delivering content and peer leadership that participants found valuable and impactful, highlighting the critical role of peer support in fostering a positive program experience.

In addition to these quantitative indicators, qualitative feedback provided further insight into the program’s acceptability and accessibility. Participants frequently expressed that the mobile-based videos and infographics were easy to understand and culturally relatable, especially when delivered in their preferred language. The flexibility of accessing content on their own time enhanced usability, particularly for those balancing work and caregiving responsibilities. Peer-led sessions were viewed as a source of emotional encouragement and accountability, with many participants stating that shared experiences made the content more trustworthy and motivating. While some initially faced challenges with digital navigation, these were largely mitigated by the simple interface and peer assistance. Overall, these perspectives affirm the intervention’s relevance, usability, and engagement value in a resource-limited setting.

### Limitations

Despite its strengths, this study has certain limitations. First, the small sample size (n=30) limits the statistical power and generalizability of the findings. As a feasibility study, the results should be interpreted as exploratory rather than conclusive. Second, the absence of a control group in this process evaluation limits the ability to attribute observed improvements solely to the MYCardio-PEER intervention. Participation in any health intervention, particularly when it includes biometric screening and feedback, can itself lead to reductions in blood pressure through increased health awareness or initiation of antihypertensive therapy for ethical reasons. While a separate manuscript reports findings from the quasi-experimental study that included a control group, this paper focuses specifically on the intervention group to assess feasibility, acceptability, and preliminary changes.

Third, data collection relied on self-reported dietary intake and physical activity, which may introduce response bias and social desirability bias. Fourth, the process evaluation was conducted in a single community in Kedah, Malaysia, which may limit the transferability of findings to other populations or regions. Finally, the relatively short program duration (8 weeks) limits insights into the long-term sustainability of the observed improvements in CVD risk factors and health behaviors. Future research addressing these limitations is warranted.

### Future Directions

While the MYCardio-PEER program demonstrated feasibility and promising improvements in cardiovascular-related behaviors, future studies are needed to build upon these findings. Larger-scale trials with control groups should be conducted to confirm the intervention’s effectiveness across diverse populations. Additionally, extended follow-up periods would help determine the sustainability of behavioral changes and the long-term impact on CVD risk factors. Future adaptations could also explore integrating health professionals or community health volunteers to enhance peer support and program scalability within different low-resource settings.

### Conclusions

This study developed and content-validated MYCardio-PEER, a peer-led, digital health lifestyle intervention tailored for a low-income community at risk of CVD. Process evaluation showed good feasibility and participant acceptability, with encouraging trends in cardiovascular-related behaviors and selected health indicators, including increased physical activity, improved dietary intake, and reductions in SBP and cholesterol levels. These findings support the feasibility and potential of integrating peer support and culturally adapted digital content in community-based CVD prevention efforts.

## Appendix

Multimedia Appendix 1 Development process of the MYCardio-PEER intervention.

Multimedia Appendix 2 Structured questionnaire for data collection.

Multimedia Appendix 3 MYCardio-PEER evaluation form.

## Abbreviations

B40: bottom 40%
CVD: cardiovascular disease
CVI: Content Validity Index
FRS: Framingham General CVD Risk Score
LDL-C: low-density lipoprotein cholesterol
MRC: Medical Research Council
SBP: systolic blood pressure
TC: total cholesterol

## Data Availability

The datasets generated or analyzed during this study are not publicly available due to ethical restrictions and the need to protect participant confidentiality, but are available from the corresponding author on reasonable request.
