# Supplementary material for: Peer-Led Digital Health Lifestyle Intervention in a Low-Income Community at Risk for Cardiovascular Disease (MYCardio-PEER): Mixed Methods Development and Process Evaluation Study
Source: J Med Internet Res. 2025 Nov 12;27:e77063. doi: 10.2196/77063 (PMC12658398; doi:10.2196/77063)
Supplement: Multimedia Appendix 1 [file jmir_v27i1e77063_app1.docx]

## ****Multimedia Appendix 1. Development process of the MYCardio-PEER intervention****

**Step 1: Review of Research Evidence**

An extensive review of published literature and national guidelines was conducted to identify effective components of peer-led lifestyle interventions for CVD prevention. This included a systematic review on peer-led interventions [1], Malaysia's Clinical Practice Guidelines on CVD prevention [2], and the Malaysian Dietary Guidelines 2020 [3]. Current review articles on digital health approaches for cardiometabolic disease were also reviewed [4-6].

**Step 2: Theoretical Framework**

The Integrated Theory of Health Behavior Change [7] guided the intervention design, focusing on knowledge acquisition, self-regulation, and social facilitation. This theory supports the inclusion of digital health tools and peer support to promote sustained behavior change [8-9]. The theoretical model is illustrated in **Figure 2**.

**Step 3: Intervention Planning**

Intervention mapping principles were applied to translate theoretical determinants into practical strategies. A behavior change matrix (Table MA1) was used to align specific objectives with intervention components. The Peer for Progress Program Development Guide [10] provided additional guidance for comprehensive peer support integration. The overall development process is summarized in Figure MA1.

Table S1. Sample of the behavioural change matrix for the development MYCardio-PEER.

| **Behavioural Theme** | **Behavioural Objectives** | **Behavioural determinants** | | |
| --- | --- | --- | --- | --- |
|  |  | **Knowledge** | **Perceived benefits** | **Self-efficacy skills** |
| Management of CVD risk factors | 1. Reduce the prevalence of modifiable CVD risk factors. 2. Promote awareness and understanding of the importance of managing CVD risk factors for cardiovascular health. 3. Encourage the adoption of healthy behaviours and lifestyle modifications to mitigate CVD risk factors. | 1. Identify the modifiable risk factors associated with CVD, such as smoking, unhealthy diet, physical inactivity, high blood pressure, and obesity. 2. Understand the link between these risk factors and the development of CVD. | - 1. Managing CVD risk factors can reduce the risk of heart disease, stroke, and other cardiovascular complications.   2. Highlight the positive impact of behaviour change on overall health, quality of life, and longevity. | - - 1. Build self-confidence and belief in one's ability to adopt and maintain healthy behaviours for managing CVD risk factors.     2. Obtain strategies and resources for smoking cessation, healthy eating, regular physical activity, blood pressure control, and weight management.     3. Enhance skills in goal setting, action planning, self-monitoring, and problem-solving to support behaviour change. |


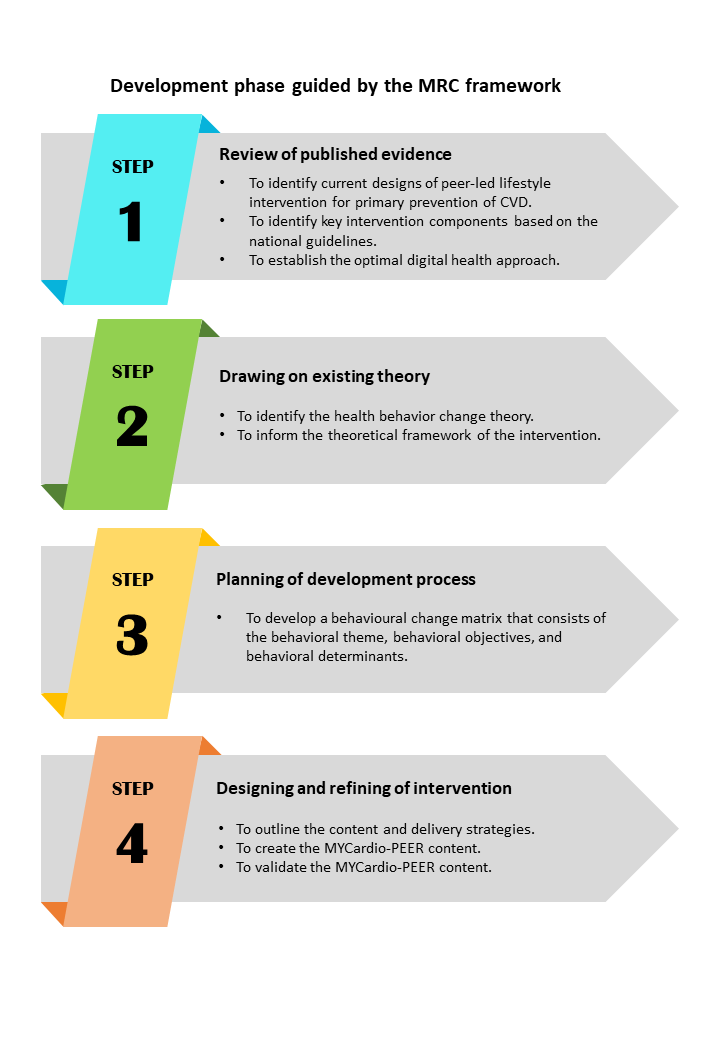


**Figure. MA1** Flow chart of the development of MYCardio-PEER

**References**

1. Lim GP, Appalasamy JR, Ahmad B, Quek KF, Ramadas A. Peer-led lifestyle interventions for the primary prevention of cardiovascular disease in community: a systematic review of randomised controlled trials. BMC Public Health. 2024;24(1):812. doi:10.1186/s12889-024-18328-w
2. Ministry of Health Malaysia. Clinical practice guidelines on primary & secondary prevention of cardiovascular disease. 2017. Accessed December 2, 2024. <https://www.moh.gov.my/moh/resources/Penerbitan/CPG/CARDIOVASCULAR/3.pdf>
3. National Coordinating Committee on Food and Nutrition. Malaysian Dietary Guidelines 2020. Ministry of Health Malaysia; 2021. Accessed December 4, 2024. <https://hq.moh.gov.my/nutrition/wp-content/uploads/2024/03/latest-01.Buku-MDG-2020_12Mac2024.pdf>
4. Liang F, Yang X, Peng W, et al. Applications of digital health approaches for cardiometabolic diseases prevention and management in the Western Pacific region. Lancet Reg Health West Pac. 2024;43:100817. doi:10.1016/j.lanwpc.2023.100817
5. Roy R, Malloy J. Evolving role of social media in health promotion. In: Health Promotion-Principles and Approaches. IntechOpen; 2023. doi:10.5772/intechopen.111967
6. Lim GP, Appalasamy JR, Ahmad B, Quek KF, Ramadas A. Social media-delivered lifestyle interventions among individuals living with diabetes and prediabetes: a scoping review. Curr Nutr Rep. 2023;12(4):721-732. doi:10.1007/s13668-023-00507-7
7. Ryan P. Integrated theory of health behavior change: background and intervention development. Clin Nurse Spec. 2009;23(3):161-170. doi:10.1097/NUR.0b013e3181a42373
8. Dunkley AJ, Tyrer F, Doherty Y, et al. Development of a multi-component lifestyle intervention for preventing type 2 diabetes and cardiovascular risk factors in adults with intellectual disabilities. J Public Health. 2018;40(2):e141-e150. doi:10.1093/pubmed/fdx067
9. Morrison LG. Theory-based strategies for enhancing the impact and usage of digital health behaviour change interventions: a review. Digit Health. 2015;1:2055207615595335. doi:10.1177/2055207615595335
10. Peer for Progress. Program Development Guide: A Resource for Developing and Implementing Peer Support Programs. 2015. Accessed December 12, 2024. <https://peersforprogress.org/wp-content/uploads/sites/1323/2024/05/PfP-Program-Development-Guide-June-2015.pdf>
