# Supplementary material for: Peer-Led Digital Health Lifestyle Intervention in a Low-Income Community at Risk for Cardiovascular Disease (MYCardio-PEER): Mixed Methods Development and Process Evaluation Study
Source: J Med Internet Res. 2025 Nov 12;27:e77063. doi: 10.2196/77063 (PMC12658398; doi:10.2196/77063)
Supplement: Multimedia Appendix 2 [file jmir_v27i1e77063_app2.docx]

Date:

Enumerator:

**Peer-led digital health framework in a B40 community at risk for cardiovascular diseases**

| InstructionsAll parts of the questionnaire should be completed.Please put a tick (√) or a cross (X) against the participant’s response, unless it is indicated otherwise.Spaces are provided for you to write their additional views on the subject. Should you need more space to write, please feel free to attach additional sheets. *Only the Investigators of the project will have access to the data.*  *All information provided here will otherwise remain confidential.*  Thank you. | Checklist (*for office use only)* | | |
| --- | --- | --- | --- |
|  | A | Sociodemographic information |  |
|  | B | Anthropometry |  |
|  | C | Medical history |  |
|  | D | Physical & clinical markers |  |
|  | E | MUSE |  |
|  | F | ABCD-M |  |
|  | G | IPAQ |  |
|  | H | Lifestyle habits |  |
|  | I | PSS-10 |  |
|  | K | Short FFQ |  |
|  | M | Edible oil use |  |
|  | L | 24-hour dietary recall |  |
|  | M | Framingham risk score |  |

| Name: |  |
| --- | --- |
|  | |
| Address: |  |
|  |  |
| Contact: |  |
|  |  |

| 1. **SOCIODEMOGRAPHIC INFORMATION** | | | | | | | | |
| --- | --- | --- | --- | --- | --- | --- | --- | --- |
|  |  | |  |  |  |  |  | |
| 1. | Age: | _______________ years | |  | 5. | Occupation: | ______________________________ | |
|  |  |  | |  |  |  |  | |
| 2. | Sex: | ☐ (1) Male  ☐ (2) Female | |  | 6. | Education: | ☐ (1) No formal education  ☐ (2) Primary school  ☐ (3) Secondary school  ☐ (4) Certificate/ diploma  ☐ (5) Bachelor  ☐ (6) Master  ☐ (7) PhD | |
|  |  |  | |  |  |  |  | |
| 3. | Ethnicity: | ☐ (1) Malay  ☐ (2) Chinese  ☐ (3) Indian  ☐ (4) Others:__________________ | |  | 7. | Personal income / month (RM): | _____________________________ | |
|  |  |  | |  |  |  |  |  |
| 4. | Marital status: | ☐ (1) Single  ☐ (2) Living with a partner  ☐ (3) Married  ☐ (4) Widowed / separated / divorced | |  | 8. | Household income / month (RM): | ______________________________ | |

| 1. **ANTHROPOMETRY** | | | |
| --- | --- | --- | --- |
|  |  |  |  |
|  |  | **First reading** | **Second reading** |
| 1. | Height (m) |  |  |
| 2. | Weight (kg) |  |  |
| 3. | Waist circumference (cm) |  |  |
| 4. | Hip circumference (cm) |  |  |
| 5. | Body fat % |  |  |
|  |  |  |  |
| 1. **PHYSICAL & CLINICAL MEASURES** | | | |
|  |  |  |  |
| 1. | Blood pressure (mmHg) |  |  |
| 2. | Fasting blood glucose (mmol/L) |  | |
| 3. | Total cholesterol (mmol/L) |  | |
| 4. | HDL-C (mmol/L) |  | |
| 5. | LDL-C (mmol/L) |  | |
| 6. | Triglyceride (mmol/L) |  | |

| 1. **MEDICAL HISTORY** | | | | | | | | | | | |
| --- | --- | --- | --- | --- | --- | --- | --- | --- | --- | --- | --- |
| 1. | Have you ever been told by a doctor that you have any of these health conditions? | | | | | | ☐ (1) Diabetes  ☐ (2) High blood pressure  ☐ (3) High cholesterol | | | | |
|  |  | | | | | |  | | | | |
| 2. | Are you taking any medications for any of these health conditions? | | | | | | ☐ (1) Diabetes  ☐ (2) High blood pressure  ☐ (3) High cholesterol | | | | |
|  |  | | | | | |  | | | | |
| 3. | Are you on a special diet? | | | | | | ☐ (1) Yes : _______________________  ☐ (2) No | | | | |
|  |  | | | | | |  | | | | |
| 4. | Within the last 6 months, have you changed your diet or lifestyle based on your doctor’s advice? | | | | | | ☐ (1) Yes ☐ (2) No | | | | |
|  |  | | | | | |  | | | | |
| 5. | Within the last 6 months, have you participated in any research that prescribed any medications or lifestyle changes? | | | | | | ☐ (1) Yes ☐ (2) No | | | | |
|  |  | | | | | |  | | | | |
| 6. | Do any of your family members have the following conditions? | | | | | |  |  |  |  | |
|  |  | | **Father** | **Mother** | **Siblings** | |  |  |  |  | |
|  | 1. Obesity: | |  |  |  | |  |  |  |  | |
|  | 1. Diabetes: | |  |  |  | |  |  |  |  | |
|  | 1. High blood pressure: | |  |  |  | |  |  |  |  | |
|  | 1. High cholesterol: | |  |  |  | |  |  |  |  | |
|  | 1. Heart disease: | |  |  |  | |  |  |  |  | |
|  |  | |  |  |  | |  |  |  |  | |
| 1. **MEDICATION UNDERSTANDING & TAKING SELF-EFFICACY QUESTIONNAIRE** | | | | | | | | | | |  |
| I am going to read you 8 statements to see how confident you are at taking/understanding your medication.  Please tell me if you strongly disagree, slightly disagree, slightly agree, or strongly agree.  **You can skip this section if you are not taking any medication.** | | | | | | | | | | |  |
| **No.** | | **Item** | | | | **Strongly disagree** | | **Slightly disagree** | **Slightly agree** | **Strongly agree** |  |
| 1. | | It is easy for me to take my medicine on time. | | | | 1 | | 2 | 3 | 4 |  |
| 2. | | It is easy for me to ask my pharmacist questions about my medicine. | | | | 1 | | 2 | 3 | 4 |  |
| 3. | | It is easy for me to understand my pharmacist’s instructions for my medicine. | | | | 1 | | 2 | 3 | 4 |  |
| 4. | | It is easy for me to understand instructions on medicine bottles. | | | | 1 | | 2 | 3 | 4 |  |
| 5. | | It is easy for me to get all the information I need about my medicine. | | | | 1 | | 2 | 3 | 4 |  |
| 6. | | It is easy to remember to take all my medicines. | | | | 1 | | 2 | 3 | 4 |  |
| 7. | | It is easy for me to set a schedule to take my medicines each day. | | | | 1 | | 2 | 3 | 4 |  |
| 8. | | It is easy for me to take my medicines every day. | | | | 1 | | 2 | 3 | 4 |  |

| 1. **ATTITUDES & BELIEFS ABOUT CARDIOVASCULAR DISEASE** | | | | | | | | | |
| --- | --- | --- | --- | --- | --- | --- | --- | --- | --- |
|  |  |  | | |  | | | |  |
| **No.** | **Item** | | | **True** | | | | **False** | |
| 1. | One of the main causes of heart attack and stroke is stress. | | |  | | | |  | |
| 2. | Walking and gardening are considered types of exercise that can lower the risk of having a heart attack or stroke. | | |  | | | |  | |
| 3. | Moderately intense activity of 2 ½ hours a week will reduce your chances of having a heart attack or stroke. | | |  | | | |  | |
| 4. | People who have diabetes are at higher risk of having a heart attack or stroke. | | |  | | | |  | |
| 5. | Managing your stress levels will help you to manage your blood pressure. | | |  | | | |  | |
| 6. | Drinking high levels of alcohol can increase your cholesterol and triglyceride levels. | | |  | | | |  | |
| 7. | HDL refers to ‘good’ cholesterol, and LDL refers to ‘bad’ cholesterol. | | |  | | | |  | |
| 8. | A family history of heart disease is not a risk factor for high blood pressure. | | |  | | | |  | |
|  |  | | |  | | | |  | |
|  |  | **Strongly disagree** | **Disagree** | | | **Agree** | **Strongly agree** | | **N/A** |
| 9. | I feel I will suffer from a heart attack or stroke sometime during my life. |  |  | | |  |  | |  |
| 10. | It is likely that I will suffer from a heart attack or stroke in the future. |  |  | | |  |  | |  |
| 11. | It is likely that I will have a heart attack or stroke some time during my life. |  |  | | |  |  | |  |
| 12. | There is a good chance I will experience a heart attack or stroke in the next 10 years. |  |  | | |  |  | |  |
| 13. | My chances of suffering from a heart attack or stroke in the next 10 years are great. |  |  | | |  |  | |  |
| 14. | It is likely I will have a heart attack or stroke because of my past and/or present behaviours. |  |  | | |  |  | |  |
| 15. | I am not worried that I might have a heart attack or stroke. |  |  | | |  |  | |  |
| 16. | I am concerned about the likelihood of having a heart attack or stroke in the near future. |  |  | | |  |  | |  |
| 17. | When I exercise for at least 2½ hours a the week I am doing something good for the health of my heart |  |  | | |  |  | |  |
| 18. | I am confident that I can maintain a healthy weight by exercising at least 2½ hours a week within the next two months. |  |  | | |  |  | |  |
| 19. | When I eat at least five portions of fruit and vegetables a day I am doing something good for the health of my heart. |  |  | | |  |  | |  |
| 20. | Increasing my exercise to at least 2½ hours a week will decrease my chances of having a heart attack or stroke. |  |  | | |  |  | |  |
| 21. | I am thinking about exercising at least 2½ hours a week. |  |  | | |  |  | |  |
| 22. | I intend or want to exercise at least 2½ hours a week. |  |  | | |  |  | |  |
| 23. | I am not thinking about exercising for 2½ hours a week. |  |  | | |  |  | |  |
| 24. | I am confident that I can eat at least five portions of fruit and vegetables per day within the next two months. |  |  | | |  |  | |  |
| 25. | I am thinking about eating at least five portions of fruit and vegetables a day. |  |  | | |  |  | |  |
| 26 | I am not thinking about eating at least five portions of fruit and vegetables a day. |  |  | | |  |  | |  |

| 1. **PHYSICAL ACTIVITY** | | | | | |
| --- | --- | --- | --- | --- | --- |
|  |  | |  | | |
| The questions will ask you about the time you spent being physically active in the **last 7 days**. . | | | | |  |
|  |  |  | | |  |
| *Think about all the* ***vigorous*** *activities that you did in the* ***last 7 days****.* ***Vigorous*** *physical activities refer to activities that take hard physical effort and make you breathe much harder than normal. Think only about those physical activities that you did for at least 10 minutes at a time.* | | | | |  |
|  |  |  | | |  |
|  |  |  | | |  |
| 1. | During the last 7 days, how many days did you do **vigorous** physical activities such as heavy lifting, digging, aerobics, or fast bicycling, or outdoor games (in days/week)? | ☐ ________days/week  ☐ (0) None *(skip to Q3)* | | |  |
|  | | | | |  |
|  | | | | |  |
| 2. | How much time did you spend doing vigorous physical activities on one of those days (in minutes/day)? | ☐ ________mins/day  ☐ (0) Don’t know/not sure | | |  |
|  |  |  | | |  |
| *Think about all the* ***moderate*** *activities that you did in the* ***last 7 days****.* ***Moderate*** *activities refer to activities that take moderate physical effort and make you breathe somewhat harder than normal. Think only about those physical activities that you did for at least 10 minutes at a time.* | | | | |  |
|  |  |  | | |  |
|  |  |  | | |  |
| 3. | During the last 7 days, on how many days did you do **moderate** physical activities like carrying light loads, bicycling at a regular pace, or doubles tennis? (Do not include walking). | ☐ ________days/week  ☐ None *(skip to Q5)* | | |  |
|  | | | | | |
|  | | | | | |
| 4. | How much time did you spend doing moderate physical activities on one of those days (in minutes/day)? | ☐ ________mins/day  ☐ (0) Don’t know/not sure | |  |  |
|  |  |  | |  |  |
|  |  |  | |  |  |
| *Think about the time you spent* ***walking*** *in the* ***last 7 days****. This includes at work and at home, walking to travel from place to place, and any other walking that you might do solely for recreation, sport, exercise, or leisure.* | | | |  |  |
|  |  |  | |  |  |
|  |  |  | |  |  |
| 5. | During the last 7 days, on how many days did you **walk** for at least 10 minutes at a time? | ☐ ________days/week  ☐ (0) None *(skip to Q7)* | |  |  |
|  |  |  | |  |  |
| 6. | How much time did you usually spend **walking** on one of those days (in minutes/day)? | ☐ ________mins/day  ☐ (0) Don’t know/not sure | |  |  |
|  |  |  | |  |  |
| *The last question is about the time you spent* ***sitting*** *on weekdays during the* ***last 7 days****. Include time spent at work, at home, while doing course work and during leisure time. This may include time spent sitting at a desk, visiting friends, reading, or sitting or lying down to watch television.* | | | |  |  |
| 7. | During the last 7 days, how much time did you spend **sitting** on a week day (in minutes/day)? | ☐ ________mins/day  ☐ Don’t know/not sure | |  |  |

| 1. **LIFESTYLE HABITS** | | | |
| --- | --- | --- | --- |
|  | | | |
| 1. | Do/did you smoke? | ☐ (1) Non-smoker *(skip to Q4)*  ☐ (2) Past smoker  ☐ (3) Current smoker |  |
|  |  |  |  |
| 2. | How long have you been smoking? | ____________________ years |  |
|  |  |  |  |
| 3. | How many cigarettes do/did you smoke per day? | ____________________ sticks |  |
|  |  |  |  |
| 4. | Do/did you drink alcohol? | ☐ (1) Non-drinker *(skip to Q6)*  ☐ (2) Past drinker  ☐ (3) Current drinker |  |
|  |  |  |  |
| 5. | How long have you been consuming alcohol? | ____________________ years |  |
|  |  |  |  |
| 6. | What time do you usually go to bed at night on weekdays? | ____________________pm / am |  |
|  |  |  |  |
| 7. | What time do you usually go to bed at night on weekends? | ____________________pm / am |  |
|  |  |  |  |
| 8. | What time do you usually wake up on weekdays? | ____________________am / pm |  |
|  |  |  |  |
| 9. | What time do you usually wake up on weekends? | ____________________am / pm |  |
|  |  |  |  |
| 10. | Do you work night shifts? | ☐ (1) Yes ☐ (2) No |  |

| 1. **STRESS** | | | | | | |
| --- | --- | --- | --- | --- | --- | --- |
| The questions in this scale ask you about your feelings and thoughts during THE LAST MONTH. In each case, please indicate your response by circle representing HOW OFTEN YOU FELT or thought a certain way.  0 Never 1 Almost Never 2 Sometimes 3 Fairly often 4 Very often | | | | | | |
|  | | | | | | |
| **In last month, how often…** | | | | | | |
| **No.** | **Item** |  |  |  |  |  |
| 1. | You been upset because of something that happened unexpectedly? | 0 | 1 | 2 | 3 | 4 |
| 2. | You felt that you were unable to control the important things in your life? | 0 | 1 | 2 | 3 | 4 |
| 3. | You felt nervous and ‘stressed’? | 0 | 1 | 2 | 3 | 4 |
| 4. | You felt confident about your ability to handle your personal problems? | 0 | 1 | 2 | 3 | 4 |
| 5. | You felt that things were going your way? | 0 | 1 | 2 | 3 | 4 |
| 6. | You found that you could not cope with all the things that you had to do? | 0 | 1 | 2 | 3 | 4 |
| 7. | Have you been able to control irritations in your life? | 0 | 1 | 2 | 3 | 4 |
| 8. | You felt that you were on top of things? | 0 | 1 | 2 | 3 | 4 |
| 9. | You been angered because of things that were outside your control? | 0 | 1 | 2 | 3 | 4 |
| 10. | You felt difficulties were piling up so high that you could not overcome them? | 0 | 1 | 2 | 3 | 4 |

| 1. **SHORT FOOD FREQUENCY QUESTIONNAIRE** | | | | | | | |
| --- | --- | --- | --- | --- | --- | --- | --- |
| These are foods typically eaten in Malaysia. Tell us how frequently you eat each of the food items (eg. if you eat rice twice a day, write 2 in the “daily” column and leave “weekly” and “monthly” columns empty), and how many servings you normally will eat (eg. 2 ladles). | | | | Use the following household measurements:  1 cup = 250ml  1 glass = 200ml  1 tablespoon = 15ml  1 dessert spoon = 10ml  1 teaspoon = 5ml | | | |
| An example is given below.  If you usually eat 4 slices of bread (any kind) 3 times in a week: | | | | | | | |
| **Food item** | | **Standard serving size** | **How many servings do you typically eat this food?** | | **How frequently do you eat this food?** | | |
|  |  |  |  |  | ***Daily*** | ***Weekly*** | ***Monthly*** |
| Bread | | 2 slices | 4 slices | |  | 3 |  |
|  | |  |  | |  |  |  |
|  | |  |  | |  |  |  |
| **Food item** | | **Standard serving size** | **How many servings do you typically eat this food?** | | **How frequently do you eat this food?** | | |
|  |  |  |  |  | ***Daily*** | ***Weekly*** | ***Monthly*** |
| 1. | **CEREAL & CEREAL-BASED PRODUCTS** | | | | | | |
| 1.1 | Rice (All cooked rice varieties – eg. white, brown, Ponni, basmati, multigrain, glutinous & parboiled rice) | 1 cup  *OR*  2 scoops |  | |  |  |  |
| 1.2 | Rice dishes (eg. Nasi lemak, nasi ayam, briyani & fried rice) | 1 cup  *OR*  2 ladles |  | |  |  |  |
| 1.3 | Rice porridge | 2 cups |  | |  |  |  |
| 1.4 | Rice noodles, laksa (fried, soup) | 1 ½ cups |  | |  |  |  |
| 1.5 | Yellow / wheat noodles (fried, soup) | 1 cup  *OR*  2 ladles |  | |  |  |  |
| 1.6 | Instant noodles | 1 packet |  | |  |  |  |
| 1.7 | Pasta, spaghetti | 1 ½ cup |  | |  |  |  |
| 1.8 | Ready-to-eat cereals (eg.cornflakes), oats | 1 cup |  | |  |  |  |
| 1.9 | Bread, slices (all varieties) | 2 slices |  | |  |  |  |
| 1.10 | Sweet bun (all varieties) | 2 small buns / 1 medium-sized |  | |  |  |  |
| 1.11 | Thosai, roti canai (all varieties) | 1 piece |  | |  |  |  |
| 1.12 | Idli, putu mayang/idiyappam | 2 pieces |  | |  |  |  |
| 1.13 | Capati | ½ piece |  | |  |  |  |
| 1.14 | Potatoes(all varieties eg. potatoes, sweet potatoes, & yam) | 1 cup diced |  | |  |  |  |
| 1.15 | Biscuits (eg. crackers) | 4 pieces |  | |  |  |  |
| 1.16 | Others: |  |  | |  |  |  |
| **Food item** | | **Standard serving size** | **How many servings do you typically eat this food?** | | **How frequently do you eat this food?** | | |
|  |  |  |  |  | ***Daily*** | ***Weekly*** | ***Monthly*** |
| **2.** | **FRUITS** | | | | | | |
| 2.1 | Fresh whole fruits (eg, apple, orange, guava) | 1 whole |  | |  |  |  |
| 2.2 | Fresh sliced fruits (eg. papaya, watermelon) | 1 piece |  | |  |  |  |
| 2.3 | Durian | 3 pieces |  | |  |  |  |
| 2.4 | Grapes | 8 pieces |  | |  |  |  |
| 2.5 | Fresh juice | 1 glass |  | |  |  |  |
| 2.6 | Others: |  |  | |  |  |  |
| **3.** | **VEGETABLES** | | | | | | |
| 3.1 | Green leafy vegetables | ½ cup |  | |  |  |  |
| 3.2 | Green raw vegetables | 1 cup |  | |  |  |  |
| 3.3 | Fruit vegetables (eg. tomato, brinjal, pumpkin) | ½ cup |  | |  |  |  |
| 3.4 | Leguminous vegetables (long beans, French beans) | ½ cup |  | |  |  |  |
| 3.5 | Cruciferous vegetables (cauliflower, broccoli) | ½ cup |  | |  |  |  |
| 3.6 | Roots (beetroot, carrot) | ½ cup |  | |  |  |  |
| 3.7 | Mushroom (all varieties) | ½ cup |  | |  |  |  |
| 3.8 | Mixed vegetables | ½ cup |  | |  |  |  |
| 3.9 | Others: |  |  | |  |  |  |
| **4.** | **LEGUMES, SEEDS & NUTS** | | | | | | |
| 4.1 | Dhal & legumes (all varieties), no gravy | 1 cup |  | |  |  |  |
| 4.2 | Nuts, seeds (all varieties) | 2 dessert spoons |  | |  |  |  |
| 4.3 | Soy milk | 1 ½ cup |  | |  |  |  |
| 4.4 | Tauhu (hard, fried, egg) | 1 ½ pieces |  | |  |  |  |
| 4.5 | Tauhu (soft) | 1 box |  | |  |  |  |
| 4.6 | Tempe | 2 pieces |  | |  |  |  |
| 4.7 | Others: |  |  | |  |  |  |
| **5.** | **MILK & MILK PRODUCTS** | | | | | | |
| 5.1 | Milk (all types) | 1 glass |  | |  |  |  |
| 5.2 | Milk powder (all types) | 4 dessert spoons |  | |  |  |  |
| 5.3 | Yogurt, tairu, mooru, dadih | 1 cup |  | |  |  |  |
| 5.4 | Cheese | 1 slice |  | |  |  |  |
| 5.5 | Others: |  |  | |  |  |  |
| **Food item** | | **Standard serving size** | **How many servings do you typically eat this food?** | | **How frequently do you eat this food?** | | |
|  |  |  |  |  | ***Daily*** | ***Weekly*** | ***Monthly*** |
| **6.** | **FISHES & SEAFOODS** | | | | | | |
| 6.1 | Fish (all types) | 1 medium piece |  | |  |  |  |
| 6.2 | Squid | 2 pieces |  | |  |  |  |
| 6.3 | Prawns, cockles (all varieties) | 12 pieces |  | |  |  |  |
| 6.4 | Dried/salted fish/seafoods/achovies | 2/3 cup |  | |  |  |  |
| 6.5 | Sardine fish, canned | 2/3 small can |  | |  |  |  |
| 6.6 | Others: |  |  | |  |  |  |
| **7.** | **POULTRY, MEAT & EGGS** | | | | | | |
| 7.1 | White meat (eg. chicken, duck) | 1 piece, palm size |  | |  |  |  |
| 7.2 | Red meat (eg. mutton, pork, beef) | 1 piece, palm size |  | |  |  |  |
| 7.3 | Eggs (hen, duck) | 2 whole |  | |  |  |  |
| 7.4 | Others: |  |  | |  |  |  |
|  |  |  |  | |  |  |  |
| **8.** | **PROCESSED FOODS, CONFECTIONARY & BEVERAGES** | | | | | | |
| 8.1 | Local kuih, cakes, doughnuts, pastries etc | 1 piece |  | |  |  |  |
| 8.2 | Chocolates, sweets, ice cream | 1 piece |  | |  |  |  |
| 8.3 | Sausages, burger | 1 piece |  | |  |  |  |
| 8.4 | Nuggets | 1 piece |  | |  |  |  |
| 8.5 | Pizza | 1 slice |  | |  |  |  |
| 8.6 | French fries | 1 medium pack |  | |  |  |  |
| 8.7 | Condensed milk | 1 tablespoon |  | |  |  |  |
| 8.8 | Milo, Horlicks | 1 glass |  | |  |  |  |
| 8.9 | Coffee, tea, cordial | 1 glass |  | |  |  |  |
| 8.10 | Carbonated drinks | 1 glass |  | |  |  |  |
| 8.11 | Butter, margarine spread | 1 teaspoon |  | |  |  |  |
| 8.12 | Jam, kaya spread | 1 teaspoon |  | |  |  |  |
| 8.13 | Peanut butter, chocolate spread | 1 teaspoon |  | |  |  |  |

| 1. **EDIBLE OIL USE** | | | | | | | |
| --- | --- | --- | --- | --- | --- | --- | --- |
| Check your kitchen for the type of oil used in cooking in your household and fill up the table below.  An example is given below: | | | | | | | |
| **Type of oil** | **Brand** | **How many bottles are usually purchased in a month?** | **What is the weight of the bottle?** | **How long it lasts?** | **Usage** | | |
|  |  |  |  |  | **Deep frying** | **Stir-frying** | **Drizzle** |
| Palm oil | Buruh | 2 | 3kg | 1 month | X | X |  |
|  |  |  |  |  |  |  |  |
|  |  |  |  |  |  |  |  |
| **Type of oil** | **Brand** | **How many bottles are usually purchased in a month?** | **What is the weight of the bottle?** | **How long it lasts?** | **Usage** | | |
|  |  |  |  |  | **Deep frying** | **Stir-frying** | **Drizzle** |
| Palm oil |  |  |  |  |  |  |  |
| Blended oil |  |  |  |  |  |  |  |
| Peanut oil |  |  |  |  |  |  |  |
| Coconut oil |  |  |  |  |  |  |  |
| Sunflower oil |  |  |  |  |  |  |  |
| Corn oil |  |  |  |  |  |  |  |
| Refined oil |  |  |  |  |  |  |  |
| Ghee |  |  |  |  |  |  |  |
| Vanaspati |  |  |  |  |  |  |  |
| Polybag |  |  |  |  |  |  |  |

How many times do you use the oil again for deep frying?

☐ Yes: ___ times

☐ No

| 1. **24-HOUR DIETARY RECALL** | | | | | | | | |
| --- | --- | --- | --- | --- | --- | --- | --- | --- |
| Please write down all the food, snacks or drinks you consume in a day.  Write in details the amount of the ingredients, sauce or gravy included in the food. Use additional papers if needed  **Day 1** | | | | | | | | |
| Day | Monday | Tuesday | Wednesday | | Thursday | Friday | Saturday | Sunday |
| Date |  |  |  | |  |  |  |  |
|  |  |  |  | |  |  |  |  |
| **Meal** | **Time** | **Food / drink** | | **Detailed ingredients** | | | **Household measurement** | |
| Breakfast |  |  | |  | | |  | |
| Mid-morning |  |  | |  | | |  | |
| Lunch |  |  | |  | | |  | |
| Tea-time |  |  | |  | | |  | |
| Dinner |  |  | |  | | |  | |
| Supper |  |  | |  | | |  | |
| Supplement |  |  | |  | | |  | |
| **Day 2** | | | | | | | | |
| Day | Monday | Tuesday | Wednesday | | Thursday | Friday | Saturday | Sunday |
| Date |  |  |  | |  |  |  |  |
|  |  |  |  | |  |  |  |  |
| **Meal** | **Time** | **Food / drink** | | **Detailed ingredients** | | | **Household measurement** | |
| Breakfast |  |  | |  | | |  | |
| Mid-morning |  |  | |  | | |  | |
| Lunch |  |  | |  | | |  | |
| Tea-time |  |  | |  | | |  | |
| Dinner |  |  | |  | | |  | |
| Supper |  |  | |  | | |  | |
| Supplement |  |  | |  | | |  | |

| 1. **FRAMINGHAM RISK SCORE FOR ASSESSMENT OF CVD RISK** | | | | | | | |
| --- | --- | --- | --- | --- | --- | --- | --- |
| **Estimation of 10 years CVD points for MEN** | | | | | | | |
| **Points** | **Age, yr** | **HDL-C** | **TC** | **SBP (not treated)** | **SBP (treated)** | **Smoker** | **Diabetes** |
| -2 |  | 1.6+ |  | <120 |  |  |  |
| -1 |  | 1.3-1.6 |  |  |  |  |  |
| 0 | 30-34 | 1.2-<1.3 | <4.2 | 120-129 | <120 | No | No |
| 1 |  | 0.9-<1.2 | 4.2-<5.2 | 130-139 |  |  |  |
| 2 | 35-39 | <0.9 | 5.2-<6.3 | 140-159 | 120-129 |  |  |
| 3 |  |  | 6.3-<7.4 | 160+ | 130-139 |  | Yes |
| 4 |  |  | >7.4 |  | 140-159 | Yes |  |
| 5 | 40-44 |  |  |  | 160+ |  |  |
| 6 | 45-49 |  |  |  |  |  |  |
| 7 |  |  |  |  |  |  |  |
| 8 | 50-54 |  |  |  |  |  |  |
| 9 |  |  |  |  |  |  |  |
| 10 | 55-59 |  |  |  |  |  |  |
| 11 | 60-64 |  |  |  |  |  |  |
| 12 | 65-69 |  |  |  |  |  |  |
| 13 |  |  |  |  |  |  |  |
| 14 | 70-74 |  |  |  |  |  |  |
| 15 | 75+ |  |  |  |  |  |  |
| Points allotted |  |  |  |  |  |  |  |
| *Grand total: ____________ points* | | | | | | | |
| **Estimation of 10 years CVD points for WOMEN** | | | | | | | |
| **Points** | **Age, yr** | **HDL-C** | **TC** | **SBP (not treated)** | **SBP (treated)** | **Smoker** | **Diabetes** |
| -3 |  |  |  | <120 |  |  |  |
| -2 |  | 1.6+ |  |  |  |  |  |
| -1 |  | 1.3-1.6 |  |  | <120 |  |  |
| 0 | 30-34 | 1.2-<1.3 | <4.2 | 120-129 |  | No | No |
| 1 |  | 0.9-<1.2 | 4.2-<5.2 | 130-139 |  |  |  |
| 2 | 35-39 | <0.9 |  | 140-149 | 120-129 |  |  |
| 3 |  |  | 5.2-<6.3 |  | 130-139 | Yes |  |
| 4 | 40-44 |  | 6.3-<7.4 | 150-159 |  |  | Yes |
| 5 | 45-49 |  | >7.4 | 160+ | 140-149 |  |  |
| 6 |  |  |  |  | 150-159 |  |  |
| 7 | 50-54 |  |  |  | 160+ |  |  |
| 8 | 55-59 |  |  |  |  |  |  |
| 9 | 60-64 |  |  |  |  |  |  |
| 10 | 65-69 |  |  |  |  |  |  |
| 11 | 70-74 |  |  |  |  |  |  |
| 12 | 75+ |  |  |  |  |  |  |
| Points allotted |  |  |  |  |  |  |  |
| *Grand total: ____________ points* | | | | | | | |
