# Supplementary material for: Peer-Led Digital Health Lifestyle Intervention in a Low-Income Community at Risk for Cardiovascular Disease (MYCardio-PEER): Mixed Methods Development and Process Evaluation Study
Source: J Med Internet Res. 2025 Nov 12;27:e77063. doi: 10.2196/77063 (PMC12658398; doi:10.2196/77063)
Supplement: Multimedia Appendix 3 [file jmir_v27i1e77063_app3.docx]

**Multimedia Appendix 3. MYCardio-PEER Evaluation Form**

**Instruction**: Please indicate your level of agreement with the statements listed below.

|  |  | Strongly agree | Agree | Neutral | Disagree | Strongly disagree |
| --- | --- | --- | --- | --- | --- | --- |
| 1. | The objectives of the program were clearly defined. |  |  |  |  |  |
| 2. | Participation and interaction were encouraged. |  |  |  |  |  |
| 3. | The topics covered were relevant to me. |  |  |  |  |  |
| 4. | The content was organised and easy to follow. |  |  |  |  |  |
| 5. | The materials distributed were helpful. |  |  |  |  |  |
| 6. | The peer activities were interesting and engaging. |  |  |  |  |  |
| 7. | The program objectives were met. |  |  |  |  |  |
| 8. | The peer leader was knowledgeable about the program topics. |  |  |  |  |  |
| 9. | The peer leader motivated me to change my health habits. |  |  |  |  |  |
| 10. | The peer leader was ready to help when I am in doubt. |  |  |  |  |  |
| 11. | The peer leader supported me to gain new knowledge and skills. |  |  |  |  |  |
| 12. | The peer leader was friendly and respectful when communicating. |  |  |  |  |  |
